# The effect of an app-based dietary intervention on diet-related greenhouse gas emissions – results from a randomized controlled trial

**DOI:** 10.1186/s12966-023-01523-0

**Published:** 2023-10-11

**Authors:** Stephanie Pitt, Linnea Sjöblom, Katarina Bälter, Ylva Trolle Lagerros, Stephanie E Bonn

**Affiliations:** 1 Cardiovascular and Nutritional Epidemiology, C6 Institute of Environmental Medicine (IMM), Karolinska Institutet, Stockholm, 171 77 Sweden; 2 Division of Clinical Epidemiology, Department of Medicine (Solna), Karolinska Institutet, Stockholm, 171 76 Sweden; 3 Department of Medical Epidemiology and Biostatistics, Karolinska Institutet, Stockholm, 171 77 Sweden; 4 Division of Public Health Sciences, Mälardalen University, Västerås, 722 20 Sweden; 5 Obesity Center, Academic Specialist Center, Stockholm Health Care Services, Stockholm, 1113 64 Sweden

**Keywords:** Dietary change, mHealth, Food frequency questionnaire, Greenhouse gas emissions, Climate change, Environmental impact

## Abstract

**Background:**

Dietary change towards a diet low in greenhouse gas emissions (GHGEs) can reduce climate impact and improve individual-level health. However, there is a lack of understanding if diet interventions can achieve low-GHGE diets.

**Methods:**

A randomized controlled trial was conducted to assess the effects of an app-based intervention. The intervention was designed to improve dietary intake of people with Type 2 diabetes, and was delivered via an app over 12 weeks, with each week covering one diet-related topic. Dietary intake was assessed at baseline and 3-month follow up by a 95-item food frequency questionnaire and linked to GHGE values. A total of *n* = 93 participants (n = 46 and n = 47 for the intervention and control group, respectively) were included in the analysis. Changes to GHGEs within and between the groups were analysed with inferential statistics.

**Results:**

The majority (60%) of participants were male, with a mean age of 63.2 years and body mass index of 30 kg/m^2^. At baseline, diet-related GHGEs were 4.8 and 4.9 kg CO_2_-eq/day in the intervention and control group, respectively. At 3-month follow up the corresponding GHGEs were 4.7 and 4.9 kg CO_2_-eq/day. We found no statistically significant changes to diet-related GHGEs within or between groups, or within food categories, from baseline to 3-month follow up.

**Conclusion:**

No evidence was found for the effectiveness of the app-based intervention to generate changes to diet-related GHGEs in a population of people with Type 2 diabetes. However, future interventions that target reducing meat consumption specifically may have the potential to result in a reduction of individual-level diet-related GHGEs.

**Trial registration:**

ClinicalTrials.gov, NCT03784612. Registered 24 December 2018. www.clinicaltrials.gov/ct2/show/NCT03784612.

**Supplementary Information:**

The online version contains supplementary material available at 10.1186/s12966-023-01523-0.

## Introduction

Greenhouse gas emissions (GHGEs) related to the global food system constitute one third of all anthropogenic emissions [1], and thus contribute substantially to climate change. Typical Western diets (common across Europe and North America) are characterized by a high intake of animal-based foods, and – due to the large environmental impact of rearing livestock – high diet-related GHGEs [2, 3]. Dietary change has therefore been recognized as an important factor to reduce GHGEs [4]. At the same time, an improvement in diet can also protect against non-communicable diseases and potentially prevent one in every five deaths globally [5]. In recognition of the association between a high intake of red and processed meat with both adverse health [6] and environmental outcomes, the World Health Organization recommends a predominantly plant-based diet as part of a healthy and sustainable lifestyle [7]. Therefore, dietary change towards a low-GHGE diet can contribute to both improved health and environmental outcomes [4].

Diets rich in plant-based foods are suggested not only to reduce GHGEs and to prevent disease [8], but also to be effective in disease management. For instance, plant-based diets have been found to contribute to effective management of Type 2 diabetes with improved glycaemic control and HbA1c (glycated haemoglobin) levels, reduced body weight, and improvements in quality of life and wellbeing [9–11]. Given the increasing global prevalence of Type 2 diabetes [12], and the known importance of dietary factors in management of the disease [13], improved dietary habits are essential to reduce disease burden and improve quality of life for patients. As an additional benefit, a reduction in diet-related GHGEs could also be achieved: since plant-based foods are comparably lower in GHGEs than animal-based foods, a healthy diet that focuses primarily on plant-based foods can be low in GHGEs [14]. It is therefore of interest to investigate if an intervention targeting healthy dietary habits alone, can have the potential to also change diet-related GHGEs.

Whilst numerous interventions have been designed to improve the lifestyle of people with Type 2 diabetes [15], a promising, but still under-utilized approach is mobile health (mHealth) interventions [16]. The field of mHealth is fast-growing, and mobile applications (apps), are increasingly integrated into dietary change interventions [17]. Due to the ubiquity of mobile phones, apps have a far-reaching potential in many societies around the world. As such, mHealth strategies have been recognized as a potentially useful, low-cost platform for dietary change interventions. However, relatively few dietary change apps are tested in research settings [18].

There are few studies on the effectiveness of app-based interventions to improve diet quality [19, 20], thus there is still a gap in current understanding of the effectiveness of such interventions to generate changes to diet-related GHGEs. As such, the aim of this study was to evaluate if the use of an app-based dietary change intervention targeting healthy dietary habits would result in changes to diet-related GHGEs in persons with Type 2 diabetes. Whilst the intervention did not provide specific recommendations to reduce diet-related GHGEs, recommendations on reducing animal-based foods (chiefly red and processed meat) and increasing legume consumption were included, hence it is feasible to consider that a change in diet-related GHGEs could be achieved.

## Methods

### The HAPPY trial

In this study, post-hoc analysis using data from the HAPPY (Healthy eating using APP technologY) Trial has been performed. The HAPPY Trial investigates a smartphone app-based intervention – the HAPPY app – the protocol for which is described in detail elsewhere [21], and briefly herein. The trial was a two-arm randomized controlled trial designed to evaluate the effects of an app-based healthy eating intervention in people with Type 2 diabetes. The target sample size for the HAPPY Trial was *n* = 200, calculated in order to detect a clinically significant change in HbA1c level of 4 mmol/mol, and based on achieving 80% power at 5% significance with an expected 20% drop out rate. The trial was approved by the ethics committee of the Regional Ethical Review Board, Stockholm, Sweden (2018/652 − 31; 2018/1094-32; 2018/2393-32; 2020 − 00591; 2020–07005; 2022-02557-02) and was registered at ClinicalTrials.gov (NCT03784612).

Recruitment of patients from primary healthcare centres in Stockholm, Sweden, began in January 2019 and ended in August 2022. Due to the Covid-19 pandemic, recruitment of study participants was paused during 2020–2021. Study participants received oral and written information about the trial and voluntarily gave written informed consent prior to study start. Participants were randomized 1:1, to the intervention and control group. Those in the intervention group gained access to the app-based healthy eating intervention at baseline, and also continued to receive usual care. The control group received only usual care from baseline to 3 months, after which access to the app was given. In this study, we have analysed the intervention effect on diet-related GHGEs from baseline to the 3-month (3m) follow up of the HAPPY Trial. As such, we explored the effects of the intervention from an environmental, rather than clinical perspective, which provides an additional outcome from those included in the original trial protocol.

The intervention was designed to improve overall dietary intake according to the Swedish national dietary guidelines, with a focus mainly on dietary habits, rather than intake of specific nutrients. The intervention was delivered via an app over 12 weeks, with each week covering one topic: 1) healthy food patterns, 2) vegetable intake (reoccurring again in week 5), 3) regular eating habits, 4) sugar, 6) carbohydrates, 7) wholegrains and fibres, 8) legumes, 9) saturated fat, 10) unsaturated fat, 11) salt, and 12) beverages. Of particular relevance to the study of GHGEs, were the topics of legumes (week 8) and saturated fats (week 9) which included recommendations to reduce red and processed meat. For each week, users were provided with healthy eating behaviours to perform (e.g., increase daily vegetable intake this week). For each day of the week, users were provided with an activity in the app (e.g., recipe 1 and 2 on Tuesday; evaluation on Sunday) alongside *edu-tainment* such as fun facts. This aimed to encourage daily use of the app, although there was no requirement to do so as the intervention was self-delivered and self-paced, with individual progress recorded in the app. Example screenshots of the intervention can be found elsewhere [21].

The intervention was designed to generate dietary change based on three theories central to intervention research: the health belief model [22] (individual health action is governed by perceptions of the threat of disease, benefits and barriers to complete an action, and self-efficacy to take action); stages of change model [23] (precontemplation, contemplation, preparation, action, maintenance, termination); and social cognitive theory [24] (behaviour change occurs due to reciprocal interactions between individual attitudes, self-efficacy, and social norms). In line with these theories, behaviour change techniques, such as general information, goal-setting strategies, self-monitoring, and feedback on performance were also included in the intervention.

### Data collection

A web-based questionnaire at baseline and follow up was used to assess demographic characteristics (e.g., age, sex, and education) and lifestyle factors, including an assessment of dietary intake through a validated food frequency questionnaire (FFQ) [25]. Dietary intake was also assessed using a 4-day food record. All participants attended in-person study meetings at baseline and follow up where anthropometric measurements of height, waist circumference, and weight were taken by study personnel, and recorded to the nearest 0.1 cm, 0.1 cm, and 0.1 kg, respectively. A study-specific referral prescription for blood sampling was also provided during the meetings.

### Dietary intake

Of the two methods to assess dietary intake – FFQ and 4-day food record – the FFQ data was used to study diet-related GHGEs. This was to ensure consistency across participants in terms of the foods for which a GHGE value was available, and provides insight into longer-term consumption patterns compared to the 4-day food record. The FFQ used to assess dietary intake contained 95 food and beverage items. Participants reported how often a portion of each item was consumed, which was used to determine average daily intake in grams. Variations in frequency could be reported (e.g., once per day, week, or month). To determine daily intake, all frequencies were converted to portions per day (e.g., *once per week* equated to 0.14 portions per day). When intake was reported in intervals, an average was taken (e.g., *1–3 times per week* equated to 2 portions a week, or 0.29 portions per day). To determine daily energy intake (kcal), standard portion sizes and calorie values were obtained from The Swedish Food Composition Database [26]. In the case of non-response on FFQ items (i.e., FFQ was completed, but some items were missing), no consumption was assumed. The FFQ items were subsequently categorized into one of the following: red and processed meat (grouped together to be in line with governmental-level dietary advice in Sweden), white meat, fish, dairy, starches and cereals (including e.g., grains, bread, and potatoes), fruit and vegetables, snacks and sweets, alcohol and other (including e.g., olive and cooking oils, salad dressing, sugar/honey, and dips).

### Greenhouse gas emissions values

GHGEs (expressed as kg CO_2_-equivalents (eq) per kg of food or beverage item) were obtained from secondary sources. All secondary sources had utilized lifecycle assessment (LCA) methodology, with the global warming potential of CO_2_ set to a 100-year time span. The chosen system boundary was *cradle-to-store-shelf*, which included emissions related to agriculture, processing and packaging, pre-purchase waste/by-products, and transport to the store-shelf. Emissions relating to land use change, or emissions past the store-shelf such as transport to the home, cooking, and waste management were not included. A more detailed explanation of the chosen system boundary is provided in Supplementary Material 1 (see File S1).

To maximise consistency across LCA methodologies used, two-thirds of GHGE values were obtained from one main source, ClimateHub from CarbonCloud [27]. In cases where no GHGE value was available, the following additional sources were used: Sjörs et al. for weight-changing items [28] (i.e., food items that change weight during cooking due to hydration or dehydration); Hjorth et al. for composite FFQ items [29] (i.e., food items/dishes made of multiple ingredients); Hallström et al. for alcoholic drinks [30]; and the Big Climate Database for five FFQ items that were not obtainable from the aforementioned sources [31]. Finally, Moberg et al. was used to ensure data consistency with respect to post-farm emissions [32]. Further information on all sources used is provided in Supplementary Material 1 (see File S1).

For some FFQ items, an adjustment to the reported GHGE value was necessary to ensure that the GHGE values for all FFQ items were calculated to the same system boundary (*cradle-to-store-shelf*). Adjustments were made under three circumstances. First, for seven FFQ items which were reported at *farm-gate*, adjustments were made by adding Swedish standard post-farm emissions as determined by Moberg et al. [32]. Second, adjustments to include emissions related to unavoidable waste, and to exclude emissions relating to avoidable waste, were also made using calculations from Sjörs et al. [28]. Third, adjustments to exclude emissions relating to land use change were also made, based on the amounts reported in The Big Climate Database [31]. In some instances, the GHGE values for similar FFQ items were assumed to be the same (e.g., GHGEs of *light juice* was assumed equal to *juice*). All adjustments and assumptions are reported in detail in Supplementary Material 1 (see File S1). The resulting GHGE value for each FFQ item is provided in Supplementary Material 2 (see File S2).

For each participant, daily intake (in kg) of each FFQ item was multiplied by the GHGE value determined for the item (in kg CO_2_-eq per kg of item). Total daily GHGEs for each FFQ item was determined by summing up all GHGEs for each FFQ item, per participant, per day.

### Study participants

**Fig. 1:**
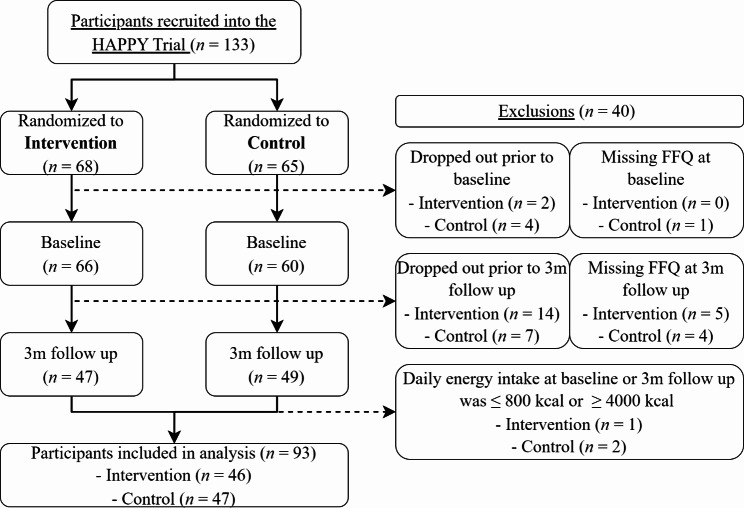
Flow diagram of participants recruited in the HAPPY Trial and included in this study

In total, 133 participants were recruited into the HAPPY Trial. In this study, we conducted a complete-case analysis, thus only participants who had completed the FFQ at both baseline and 3m follow up were included in the analytic sample. Exclusions were made of participants who dropped out prior to baseline (*n* = 6), had missing FFQ data at baseline (*n* = 1), dropped out prior to 3m follow up (*n* = 21), had missing FFQ data at 3m follow up (*n* = 9), and extreme under- and over-reporters, with a mean intake of $\le$800 or $\ge$ 4000 kcal per day (*n* = 3). Cut off points were determined based on previous literature [14]. Figure 1 shows a flow diagram of the included and excluded participants, separated by intervention group. After the exclusion of participants, a total of 93 participants with complete baseline and 3m FFQ data were included in our analyses. Background characteristics of age, sex, BMI, and physical activity levels in the final analytic sample did not differ from excluded participants who had completed baseline assessments (*p* > 0.05).

### Data analysis

All analyses were performed using Stata 15.1. Significance was set to 5% (α = 0.05). Descriptive statistics – means and standard deviations (SD) or numbers of participants (*n*) and percentages (%) – of baseline characteristics including age, sex, daily energy intake, education level, body mass index (BMI), and weekly physical activity were determined. T-tests were performed for continuous characteristics, and Chi-squared tests for categorical characteristics, to determine if there were differences between the intervention and control group at baseline.

Analyses of the intervention effect were performed using an intention-to-treat approach. Within group differences (i.e., differences within the intervention and control group separately) were determined by conducting t-tests between participant diet-related GHGEs at baseline and 3m follow up. The absolute change in daily diet-related GHGEs from baseline to 3m follow up was calculated for each participant by subtracting the baseline value from the 3m follow up value. A box-and-whisker plot was generated to depict the spread of change in daily diet-related GHGEs, by intervention group. The between group difference was determined by conducting a t-test between the mean change in daily diet-related GHGEs of both groups. To determine if any specific changes were made to the diet, FFQ item categories were analysed. The mean diet-related GHGEs for each FFQ item category at baseline and 3m follow up was determined for both groups and presented in a bar graph. Differences in mean GHGEs for each FFQ item category were determined by conducting t-tests between baseline and 3m follow up values, for the intervention and control group.

## Results

### Baseline characteristics

Of the 93 included participants, 56 were male (60%). The mean age of all participants was 63.2 years (SD 10.8). The mean BMI of participants was 30.0 kg/m^2^ (SD 5.1), with 44% of participants having a BMI $\ge$30 kg/m^2^. The mean daily energy intake was 2041 kcal/day (SD 591). The mean weekly exercise was 234 min (SD 117). The majority (70%) of participants reported completing $\ge$150 minutes per week. Most participants (57%) were well educated and had university education (*n* = 53). There were no statistically significant differences in any characteristic between the intervention and control groups (Table 1).

**Table 1:** Baseline characteristics of participants included in the analysis

Characteristic	Intervention (*n* = 46)	Control (*n* = 47)	*p value* ^+^
	Mean (SD)	Mean (SD)	
Age (years)	63.6 (10.4)	62.8 (11.3)	0.72
BMI (kg/m^2^)	30.5 (5.9)	29.6 (4.1)	0.44
Total energy intake (kcal/day)	2056 (585)	2026 (601)	0.81
Weekly physical activity (mins)	225 (124)	243 (110)	0.45
	*n* (%)	*n* (%)	
Sex			0.90
Female	18 (39)	19 (40)	
Male	28 (61)	28 (60)	
Education			0.60
Primary/Secondary school	21 (48)	17 (36)	
College/University	23 (50)	30 (64)	
Don’t know/want to answer	1 (2)	0 (0)	

SD: Standard Deviation; BMI: Body Mass Index. ^+^p-values obtained by t-test for continuous variables and by Chi-squared test for categorical variables

### Change in diet-related greenhouse gas emissions

Mean daily diet-related GHGEs at baseline and 3m follow up are shown in Table 2, by intervention group. At baseline, the mean daily diet-related GHGEs were 4.8 and 4.9 kg CO_2_-eq, for the intervention and control group, respectively. When scaled up over one year, the mean yearly diet-related GHGEs at baseline were 1.7 and 1.8 metric tonnes CO_2_-eq for the intervention and control, respectively. At 3m follow up, the mean daily diet-related GHGEs were 4.7 and 4.9 kg CO_2_-eq, for the intervention and control group, respectively. There was no statistically significant within-group difference in daily diet-related GHGEs between baseline and 3m follow up within either the intervention or control group.

The mean absolute change in daily diet-related GHGEs was − 0.1 and 0.0 kg CO_2_-eq for the intervention and control group, respectively (Table 2). Over one year, this equates to a mean reduction of approximately 35 kg CO_2_-eq per person, per year for the intervention group. There was no statistically significant difference between the intervention and control group for the absolute change in daily diet-related GHGEs.

**Table 2:** Mean diet-related GHGEs for the intervention and control group at baseline and 3m follow up, and the associated change between the two time points

	Diet-related GHGEs (kg CO_2_-eq / day) Mean (SD)	
	Intervention	Control
Baseline	4.8 (1.4)	4.9 (1.9)
3m follow up	4.7 (1.4)	4.9 (1.7)
Mean change in GHGEs	-0.1 (1.3)	0.0 (1.7)
*p value within group differences*	0.62	0.98
*p value between group difference*	0.78	

GHGEs: Greenhouse gas emissions; 3m: 3-month follow up. The within group difference is the difference in diet-related GHGEs between baseline and 3m follow up (i.e., the change) for the intervention and control group, separately. The between group difference is the difference in the mean change between the intervention and control group

**Fig. 2:**
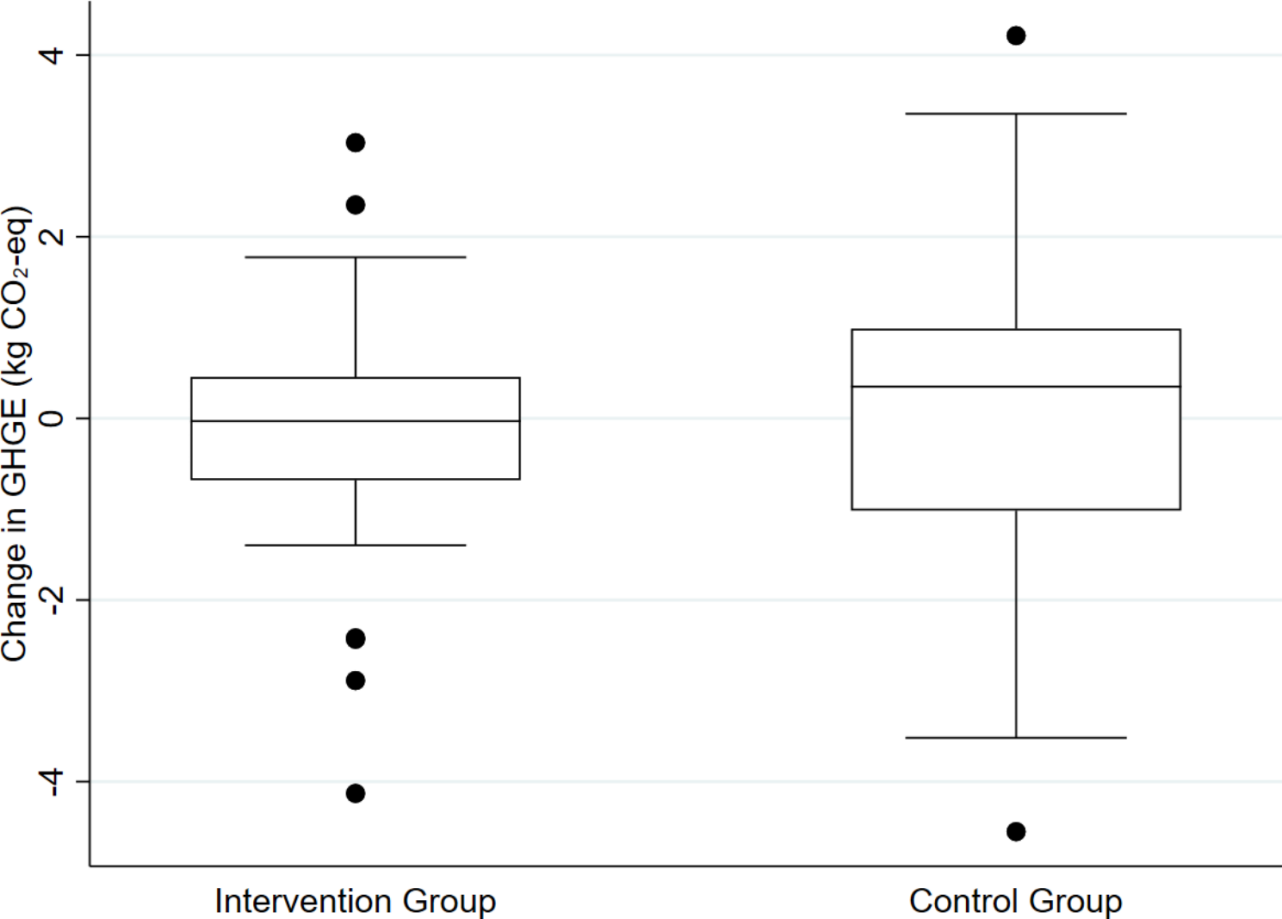
Spread of change in daily diet-related GHGEs between baseline and 3-month follow up by intervention group

The box-and-whisker plot in Fig. 2 shows the spread of change in daily diet-related GHGEs for both the intervention and control group. Much greater variation in change was found for the control group, compared to the intervention group.

### Change food category greenhouse gas emissions

**Fig. 3:**
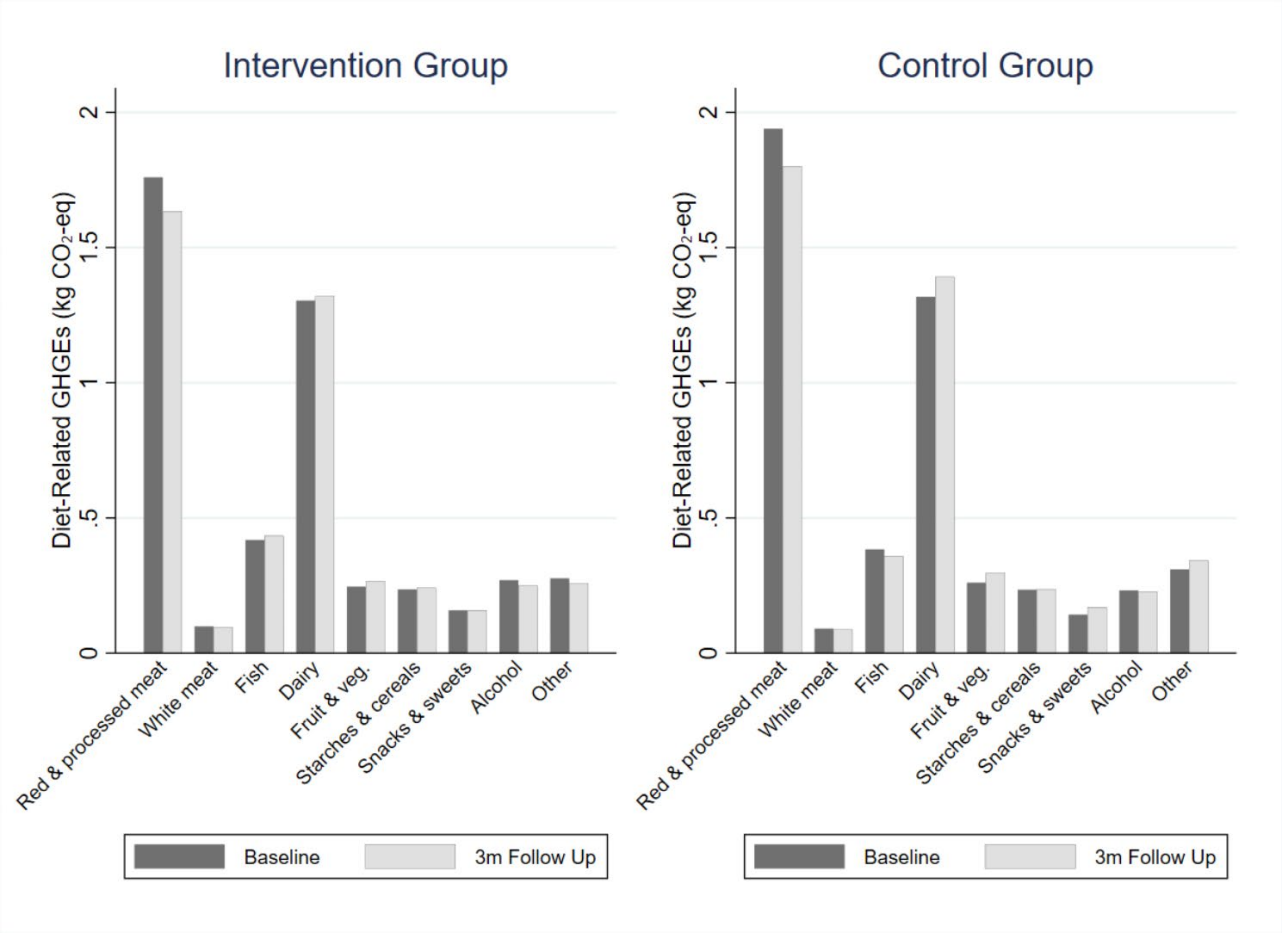
Bar graph showing the mean diet-related GHGEs associated with each FFQ item category at baseline and at 3-month follow up, by intervention group

The mean GHGE for each FFQ item category at baseline and 3m follow up is shown in Fig. 3. The greatest contributor to diet-related GHGEs in both groups was the category of red and processed meat, and then dairy products. In the intervention group, the mean daily GHGEs relating to red and processed meat consumption was 1.7 kg CO_2_-eq at baseline and 1.6 kg CO_2_-eq at 3m follow up. In the control group, the mean daily GHGEs relating to meat consumption was 1.9 kg CO_2_-eq at baseline and 1.8 kg CO_2_-eq at 3m follow up. There was no statistically significant difference between GHGEs at baseline and 3m follow up for any FFQ item category, in either the intervention or control group. For all participants at baseline, the mean consumption of red and processed meat was 83 g per day, which constituted 8% of the total mean energy intake, but contributed 41% to the mean diet-related GHGEs.

## Discussion

This study aimed to increase the understanding of the effectiveness of an app-based dietary change intervention as a method to generate changes to diet-related GHGEs. This was the first study to investigate any aspect of dietary change relating to the specific intervention. We did not observe any change in diet-related GHGEs between baseline and 3m follow up in the intervention group, and no statistically significant changes were made to diet-related GHGEs within any food category. Given that red and processed meat consumption constituted the greatest proportion to diet-related GHGEs at baseline and 3m follow up, changes to diet-related GHGEs overall may have been limited due to the small and statistically non-significant changes made in consumption of these products, specifically.

### Results in context

The mean diet-related GHGEs in this study ranged between 1.7 and 1.9 tonnes CO_2_-eq per participant per year, which is supported by both Swedish [14, 33] and European [34] literature. However, while the diet-related GHGEs found in our study align well with some studies that utilized an FFQ [14, 35], they were higher than in other studies [28, 36]. For example, despite both being in the Swedish context, the lower mean diet-related GHGEs found for participants studied by Sjörs et al. [28] could be explained by differences in study populations. Their participants were self-selected and predominantly young (mean age of 33 years), well-educated females, whereas our study population were predominantly older males (mean age of 63 years), who have been found to have higher diet-related GHGEs compared to females in Sweden [14]. In the study by Biesbroek et al. [36] participants had a wider age range (20–70 years old) than our study, and a lower mean BMI. A higher BMI has been associated with higher diet-related GHGEs [29, 37, 38].

The overall lack of consistency in diet-related GHGEs across the mentioned studies highlights that large variation in diet-related GHGEs exists, both within and between different populations. Despite diet-related GHGEs being heavily impacted by different dietary habits [39], meat consumption in this study – and in other published literature [33, 36, 40–42] – was found to constitute the largest majority to total diet-related GHGEs. Therefore, these findings indicate that there is a need to address reducing meat consumption within any intervention that aims to achieve low-GHGE diets.

When considering possible improvements to the HAPPY intervention to generate dietary change to reduce diet-related GHGEs, an increased focus on the environmental impact of food is likely needed. This could be achieved by, for example, including facts on foods low and high in GHGEs alongside those currently included, or providing an estimate of the total GHGEs within the recipe section in order to improve awareness and familiarity with the topic. In addition, including multiple components could be considered. For instance, a multi-component intervention – consisting of a group workshop and a 12-week app-based program – was evaluated in a randomized controlled trial in people with Type 2 diabetes in Spain, with a mean age of 60.6 years [43]. The study found evidence for the effectiveness of the intervention on improving dietary intake in line with a Mediterranean diet, which has been found to be lower in GHGEs compared to a traditional Western diet [44, 45]. Due to the similarity between the study populations, the results from Alonzo-Domínguez et al. [43] could be applied when considering future improvements of the HAPPY intervention, considering that non-app components may support dietary change more in older adults, who may have decreased usability of the app.

### Strengths and limitations

There are several strengths and limitations of the study methods that should be acknowledged. First, the small sample size is a limitation, reducing the statistical power to detect an effect of the intervention on diet-related GHGEs. Second, for the intervention group, change in diet-related GHGEs could be correlated to app usage (i.e., adherence to the intervention), which was assumed equal for all participants in the intervention group, but may not be. As such, it is a limitation that there was no data on participant adherence to the intervention available, as this possible explanation for the null findings cannot be excluded. However, the null findings are more likely due to the primary aim of the intervention, as it did not encourage participants to follow a low GHGE diet per se, although considerable content was provided on reducing red and processed meat intake and increasing plant-based food intake. Finally, the presented research benefits from analysing randomized data, thus reducing the effects of confounding variables.

The use of a validated FFQ to assess dietary intake is a strength. Furthermore, the FFQ included a large number of items, thus is likely to cover a considerable proportion of the diet. However, as in any study utilizing an FFQ, some limitations remain, for example the use of self-reported data and possible introduction of social desirability and/or recall bias. As such, FFQ assessments can sometimes underestimate true dietary intake. To address these limitations, missing information on portion size was added using Swedish standard values. Furthermore, any inaccuracies in assessing dietary intake are likely to occur at both baseline and 3m follow up, within both the intervention and control. Therefore, all participant dietary intake is likely subject to the same potential measurement error, thus reducing the impact of the noted limitations on the findings.

In terms of assessing the environmental impact of diet as a whole, it is important to acknowledge that GHGEs constitute only one indicator of environmental impact: land use, water use, and eutrophication potential among others are also important to consider. However, based on information available for the purposes of this study, only GHGEs have been included, thus presenting a limitation to the full assessment of environmental impact of diet. Furthermore, as with any study utilizing GHGE data, there are strengths and limitations of the methods applied in determining the GHGE value of FFQ items. In this study, GHGE values were obtained from multiple secondary sources, in which different LCA studies may have allocated GHGEs differently, hence potentially introducing some error. Such differences in GHGE values for the same food item can occur due to, for example, growing and production methods, weather and soil conditions, and transport. However, two-thirds of the GHGE values in this study were determined from one source, and the GHGE values for all FFQ items are estimated to the same system boundary. This enables accurate comparisons between different items and categories. Furthermore, using an LCA to determine GHGEs provides an estimated value, rather than a precise figure. To address this limitation, where necessary, adjustments of post-farm emissions and weight changes were made to FFQ items based on Swedish-specific standards and previous Swedish literature [28], respectively. This improves the precision of the diet-related GHGE values determined. Since the same GHGE value for each FFQ item was applied to all participants, at both baseline and 3m follow up, this limitation does not impact the findings, but can limit comparability with other studies, particularly those outside of a Swedish context. As such, it is challenging to compare GHGEs between countries. However, consumption of meat, which notably contributes to diet-related GHGEs, is high within a typical Western diet, thus the findings are likely generalizable to countries in which a Western diet is common.

### Wider implications

The category of red and processed meat was found to be the greatest contributor to diet-related GHGEs. Moreover, at baseline, participants in this study ate on average 83 g of red and processed meat per day (cooked weight), corresponding to 581 g per week. This is above the recommended 500 g limit in Sweden suggested to avoid an increased risk of adverse health outcomes [46]. As such, the findings indicate a large potential scope for change. Given that addressing high diet-related GHGEs was not the primary aim of the intervention, it remains possible that interventions specifically designed to target diet-related GHGEs may have an effect. However, within the scope of the current food system, there is a limit to how low individual diet-related GHGEs can be. Therefore, in combination with such interventions, strategies to alter the environments in which societies operate may be necessary [47]. For instance, introducing a meat tax and improved food labelling, in addition to education and individual empowerment, have been suggested as possibilities to create shifts in social norms, and hence achieve dietary change [48–50]. Allocating resources to addressing systemic problems within food systems, such as inequity, access, and affordability, are also crucial to generating more sustainable diets [51] and improving health.

## Conclusion

We found no evidence for the effectiveness of the 3-month HAPPY app-based intervention for healthy eating to generate changes to diet-related GHGEs in a population of persons with Type 2 diabetes. Nonetheless, our findings indicate there is potential to reduce diet-related GHGEs through reductions in meat intake, which could be achieved with interventions specifically developed to target this in individual-level diets.

### Electronic supplementary material

Below is the link to the electronic supplementary material.

Supplementary Material 1

Supplementary Material 2

Supplementary Material 3

Supplementary Material 4

## Data Availability

Participant data analysed in the current study are not publicly available due to ethical restrictions but are available from the principal investigator (SEB) on reasonable request. GHGE data utilised in the current study (value, adjustments, and source) for all FFQ items is provided in the Supplementary Material (SM2).

## List of abbreviations

3m: 3-month
Apps: (mobile) Applications
BMI: Body Mass Index
FFQ: Food Frequency Questionnaire
GHGE: Greenhouse gas emission
HAPPY: Healthy eating using APP technologY
HbA1c: Glycated haemoglobin
LCA: Lifecycle Assessment
mHealth: Mobile health
SD: Standard Deviations
