# Supplementary material for: The effect of an app-based dietary intervention on diet-related greenhouse gas emissions – results from a randomized controlled trial
Source: Int J Behav Nutr Phys Act. 2023 Oct 11;20:123. doi: 10.1186/s12966-023-01523-0 (PMC10568795; doi:10.1186/s12966-023-01523-0)
Supplement: Supplementary file 1 — Supplementary Material 1 [file 12966_2023_1523_MOESM1_ESM.docx]

# **Supplementary Material S1**

## 1 Life Cycle Assessment and System Boundaries

Greenhouse gas emission (GHGE) values were determined from secondary sources that had utilized a life cycle assessment (LCA) methodology. LCAs often estimate GHGEs associated with the ‘*cradle-to-grave*’ of a product (i.e., throughout the entire lifecycle). However, with food and beverage items, *cradle-to-farm* or *cradle-to-store-shelf* can also be utilized (i.e., a sum of all GHGEs from growing the item to placing it on the store shelf). The extent to which the lifecycle is covered is referred to as the system boundary. However, different LCA studies use different system boundaries, thus making it challenging to accurately compare between items. Furthermore, variations in the true GHGE value for the same item are always present due to differences in factors such as production method, soil quality, and weather.


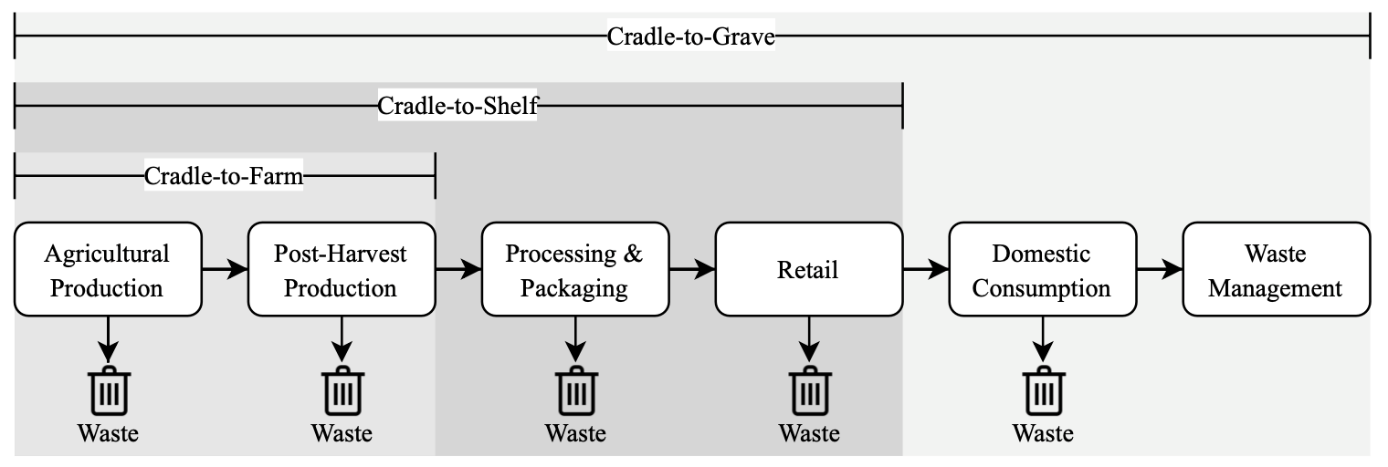
**Figure S1** shows the different phases included in each system boundary. GHGE values obtained from LCA data are approximate estimates and are not exact amounts. In our study, the chosen system boundary was *cradle-to-store-shelf*, which included emissions related to agriculture, processing and packaging, pre-purchase waste/by-products and transport to the store-shelf. Emissions related to land use change, or emissions past the store-shelf such as transport to the home, cooking, and waste management were not included.

**Figure S1:** Simplification of separate phases during the whole life cycle of a food item and system boundaries. Greenhouse gases are emitted at each stage. Adapted from [1].

## 2 Methods supplement

## *Sources of GHGE Data*

Sources of GHGE data were determined by searching the literature in PubMed and Google Scholar, and by conducting a Google search. Sources were selected for further investigation if they were open access, in the Swedish context, and had utilized an LCA to determine GHGEs.

## *Main Sources*

Relevant sources that contained GHGE values for more than 50 items were termed “main” sources and were ranked based on their recentness/frequency of update and the percentage of food items in Food Frequency Questionnaire (FFQ) covered by the source. The highest ranked main source was the ClimateHub from CarbonCloud [2], which had GHGE data on 67% of FFQ items and was updated ~3 months.

## *Additional Sources*

Furthermore, four additional sources were used for the remaining 33% FFQ items, or for making necessary adjustments to GHGE values.

1. **Sjörs *et al*.,** [3]: Used for *weight-changing* food items. Weight change occurs during cooking (e.g., hydration of pasta or rice, and dehydration of meat or fish) and due to unavoidable waste (e.g., peel and shell). This is crucial to factor in as the standard portion sizes used in the FFQ are based on cooked food, but GHGE values are based on uncooked food.
2. **Hjorth *et al.*,** [4]: Used for *composite* food items (i.e., those made of several main ingredients), which had been calculated based on the weighted averages of the three main ingredients, using standard Swedish recipes.
3. **Hallström *et al*.,** [5]: Used for *alcoholic drinks* in Sweden.
4. **The Big Climate Database** [6]: A Danish-specific source used for five “missing” FFQ items that did not have a GHGE value obtainable from any of the above Swedish sources.

## 3 Assumptions and Adjustments

Since there was not a single source that had data on all 95 food and beverage items, we used multiple sources (and thus multiple LCA studies). As such, for some FFQ items, adjustments or assumptions were necessary in order to maximize consistency.

**Adjustments** were made to the GHGE values of FFQ items under the following scenarios:

1. For seven FFQ items from ClimateHub, the GHGE values were not reported at *store-shelf*, but *farm-gate*. Therefore, adjustments were made by adding on Swedish standard post-farm emissions obtained from Moberg *et al*. [7]
2. Where necessary, GHGE values were adjusted to include emissions relating to unavoidable waste and to exclude emissions relating to avoidable waste using calculations from Sjörs *et al*. [3]. Avoidable emissions were excluded for three reasons. First, avoidable food loss (i.e., household waste) can vary greatly depending on the individual. Second, the standard portion sizes used for the FFQ do not consider uneaten food. Third, accounting for avoidable food loss goes beyond the store-shelf system boundary, and arguably into the lifecycle stage of waste management, which is not considered in the GHGE calculations for this study. However, for *composite items* from Hjorth *et al*. it was not possible to re-adjust the GHGE value to remove adjustments made of avoidable waste loss as the calculated GHGE values were not reported in enough detail to determine how the contribution of avoidable waste. The inaccuracy introduced is expected to have only a minor, if any, impact on the final assessed GHGE value. Furthermore, since this affects only six FFQ items, will likely have a minor, if any, impact on the determined diet-related GHGEs of participants.
3. GHGE values for FFQ items obtained from The Big Climate Database [6] were adjusted to exclude emissions relating to land use change, since it was not possible to consistently include such emissions across all FFQ items.

**Assumptions:** For four FFQ items, no GHGE value was obtained from the literature, hence assumptions were made based on their similarity to other items: *fruit soup* and *light juice* were assumed to have an equal GHGE value as *juice*; *cooking margarine* and *liquid margarine* were assumed equal to *margarine*; *wholewheat bread*, based on a study from Espinosa *et al*. [8], was assumed to have a 6.5% lower GHGE than *white bread*. In instances where the FFQ specified multiple food items in one question (e.g., apples/pears; nuts/almonds; or berries fresh/berries frozen), the final GHGE value was based on an average of both food items when obtainable from the same source. Finally, one item on the FFQ, *black pudding*, was excluded, as no GHGE value could be found from any of the sources.

All GHGE values for each FFQ item, along with calculations and notes, are available in Supplementary File S2.

**References**

[1] Martin, M., Oliveira, F., Dahlgren, L., & Thornéus, J. Environmental implications of Swedish food consumption and dietary choices. 2016. IVL Swedish Environmental Research Institute.

**Not referenced in main text.**

[2] CarbonCloud. ClimateHub. 2022. (Online) Available from:

<https://apps.carboncloud.com/climatehub/> [Accessed 20Jan2023].

**Reference 24 in main text**

[3] Sjörs, C.; Raposo, S. E.; Sjölander, A.; Bälter, O.; Hedenus, F.; Bälter, K. Diet-Related Greenhouse Gas Emissions Assessed by a Food Frequency Questionnaire and Validated Using 7-Day Weighed Food Records. Environ Health, 2016, 15 (1). <https://doi.org/10.1186/s12940-016-0110-7>.

**Reference 25 in main text**

[4] Hjorth, T.; Huseinovic, E.; Hallström, E.; Strid, A.; Johansson, I.; Lindahl, B.; Sonesson, U.; Winkvist, A. Changes in Dietary Carbon Footprint over Ten Years Relative to Individual Characteristics and Food Intake in the Västerbotten Intervention Programme. Sci Rep, 2020, 10 (1). <https://doi.org/10.1038/s41598-019-56924-8>.

**Reference 26 in main text**

[5] Hallström, E.; Håkansson, N.; Åkesson, A.; Wolk, A.; Sonesson, U. Climate Impact of Alcohol Consumption in Sweden. J Clean Prod, 2018, 201, 287–294.

<https://doi.org/10.1016/j.jclepro.2018.07.295>.

**Reference 27 in main text**

[6] CONCITO. The Big Climate Database Version 1 1. 2021. (Online) Available from: https://denstoreklimadatabase.dk/en/download [Accessed 20Jan2023]

**Reference 28 in main text**

[7] Moberg, E.; Walker Andersson, M.; Säll, S.; Hansson, P.-A.; Röös, E. Determining the Climate Impact of Food for Use in a Climate Tax—Design of a Consistent and Transparent Model. Int J Life Cycle Assess, 2019, 24 (9), 1715–1728. <https://doi.org/10.1007/s11367-019-01597-8>.

**Reference 29 in main text**

[8] Espinoza-Orias N, Stichnothe H, Azapagic A. The carbon footprint of bread. The International Journal of Life Cycle Assessment, 2011, Vol 16(4), 351–65.

**Not referenced in main text**
